# Miscellaneous

**Published:** 1891-03

**Authors:** 


### MISCELLANEOUS.

### TALLEYRAND.

THE publication in the *Century Magazine* of the memoirs of this noted courtier and diplomat has served to bring out some interesting anecdotes connected with his strange career, which might otherwise never have been brought to the surface. We extract the following from the *Brooklyn Eagle*, whose publication office is only a stone's throw from the old time bluff to which it refers.

When Talleyrand fled from France at the beginning of the Reign of Terror, he sought a refuge in England. There he remained unmolested until January of 1794, when, on the supposition that he was more intimately connected with the events that led to the execution of Louis XVI. than he cared to admit, he received an order to quit England, and after vainly appealing to the foreign secretary, he sailed for the United States, bearing with him letters of introduction to a number of prominent Americans, among them one from the Marquis of Lansdowne to Washington.

He was accompanied in his flight by a M. de Beaumetz, with whom, some time after their arrival in America, he formed a partnership for the purpose of fitting out a trading vessel. A small ship was freighted with goods for Calcutta, whither the two exiles had resolved to proceed in search of fortune ; and all that was needed to enable them to put their scheme into operation was a fair wind, which, however, the elements refused. In the interval caused by this detention, Talleyrand had one of what he called his presentiments, and to its occult warnings, he afterward declared, he owed the immediate preservation of his life, salvation from shipwreck, and that change in his destiny which led to all the future incidents of his eventful career. Disappointment and vexation, preying upon an irritable temper, drove his partner mad. Talleyrand saw insanity in his looks and gestures, but, suffering himself to be led to the Heights of Brooklyn, which overlook the harbor, he turned upon the maniac, and, fixing his eyes sternly upon him, exclaimed :

" Beaumetz, you mean to murder me. You intend to throw me from the Heights into the sea below. Deny it, monster, if you can."

Thus apostrophized, the unhappy and conscious-stricken maniac quailed beneath the intensity and sternness of his gaze ; confessed that such was his design ; that the thought, like a flash from the lurid fire of hell, had haunted him day and night ; and, flinging himself upon the neck of his meditated victim, he burst into tears and implored forgiveness. The paroxysm had passed off and tottering reason had resumed her sway. Beaumetz was conveyed home and placed under medical treatment, and, speedily recovering, proceeded on his voyage alone, never more to be heard of.

" My fate," said Talleyrand, when speaking of this incident in after life, "was at work."

---

GREASE ON SILK.—Grease may be removed from silk by applying magnesia to the wrong side.

### ALCOHOLIC PRESCRIPTIONS.

We clip the following from *Good Health :*

There is unquestionably much mischief done by physicians prescribing alcohol as a remedy for disease. Many physicians are in the habit of prescribing alcohol for nearly all maladies and nearly all patients indiscriminately. The writer has met a number of cases in which persons have been made confirmed drunkards by this practice. During the prevalence of the epidemic of la grippe some months ago, several instances came under our notice in which persons kept themselves in a state of semi-intoxication for days, either to prevent an attack, or as a means of curing a slight attack, of the disease. An eminent French physician publicly recommended alcohol as a preventive remedy for la grippe, in consequence of which fifteen hundred persons were arrested on the following day for drunkenness. Twelve hundred of these people declared that they had done nothing more than follow the prescription of the physician referred to.

### IDA LEWIS, THE HEROINE.

Ida Lewis, the heroine of Lime Rock lighthouse, who has saved the lives of so many persons, receives from the government a salary of $750 a year and two tons of coal. When her father became paralytic she was made custodian of the light for life. In appreciation of her heroic efforts in saving lives she has a gold medal from the United States Treasury Department, three silver medals from the State of Rhode Island, one from the Humane Society of Massachusetts, and another from the New York Life-Saving Association. It was in 1869 that Gen. Grant presented her the splendid life boat Rescue, which she now has. James Fisk, Jr., built a boat house for it, and also sent the heroine a silk flag made by Mrs. McFarland, of New York. Miss Lewis is a member of Sorosis, and was presented a gold brooch by that organization. She also has a number of valuable articles from private individuals.

### PRACTICAL RECIPES.

THE following varnish will maintain its transparency, and the metallic brilliancy of the articles will not be obscured : Dissolve ten parts of clear grains of mastic, five parts of camphor, five parts of sandarach, and five parts of elemi in a sufficient quantity of alcohol, and apply without heat.

A GOOD CEMENT for joining parts of apparatuses, etc., permanently solid and waterproof, and which resists heat, oils, and acids, is made by mixing concentrated sirupous glycerine with finely powdered litharge to a thick, viscid paste, which is applied like gypsum. Glass, metal, and wood can be cemented together by it.

IMPERMEABLE GLUE.—To make an impermeable glue, soak ordinary glue in water until it softens, and remove it before it has lost its primitive form. After this, dissolve it in linseed oil over a slow fire until it is brought to the consistence of a jelly. This glue may be used for joining any kinds of material. In addition to strength and hardness, it has the advantage of resisting the action of water.

### BUTTON BOOTS.

Why, asks a correspondent, do women who haven't got pretty feet, and who know they haven't, wear just the boots that make their feet look their very worst? A woman with big, shapeless feet, or crooked feet, can afford to wear but one kind of boot—a laced one, and never a low shoe in any circumstances. A buttoned boot does all very well for the first few days, while it still buttons trim and snug about the ankles ; but every woman knows that it does this for a few days only, then it loosens and begins to take on the shape of the foot, exaggerating its peculiarities every day just a little, and by-and-by before the boot is half worn out, it is a kind of caricature of the foot with every defect and imperfection exaggerated. The laced boot doesn't do this, because it can be drawn up every morning like a new boot, holding the foot always firmly and securely, and so acts as a corrective against any tendency the foot has to be ill-shaped and spreading.

---

### JEWISH MEAT.

A visit to a Jewish butcher's shop would probably surprise some of our physiologists and might be of great advantage to them. The Jewish butcher is not an uneducated man, like the average British knight of the pole-axe ; indeed, he does not use the pole-axe at all. He is specially trained to kill, and to kill painlessly. But what is more, at any rate from a consumer's point of view, he is specially trained to inspect the slaughtered carcass, and to inspect it accurately. When the Jewish butcher has inspected his slaughtered animals, he divides them into two classes. The one class he calls "Kosher," and the other "Trifa." Every carcass of a slaughtered animal that is perfectly free from disease of every kind, and in all its parts, he puts aside as "Kosher," or wholesome meat ; and that may be freely eaten by the Jews. Every carcass, on the other hand, that shows any indication of disease, however slight, in any of its organs, is rejected as "Trifa," and is not permitted by the Jewish law to be eaten. In ordinary cases it would have to be destroyed ; but probably in these times it may be sold to the less particular and less scientific Gentiles.

---

In scarlet fever, the skin should be daily rubbed with carbolic acid (one drachm) and vaseline (five ounces). This will not only relieve the itching, but disinfect the skin, and prevent the air from being contaminated with scales and exhalations.

A Soldier's Retort.—During the summer of 1863, while the hospitals at Canton, Miss., were crowded with sick and wounded soldiers, the ladies visited them daily, carrying with them delicacies of every kind, and did all they could to cheer and comfort the suffering. On one occasion a pretty miss of sixteen was distributing flowers and speaking gentle words of encouragement to those around her, when she overheard a soldier exclaim, "Oh, my Lord !" Stepping to his bedside to rebuke him for his profanity, she remarked, "Didn't I hear you call on the name of the Lord ? I am one of His daughters. Is there any thing I can ask Him for you ?" Looking up into her bright, sweet face, he replied, "I don't know but what there is." "Well," said she, "what is it ?" Raising his eyes to hers and extending his hand, he said, "Please ask Him to make me His son-in-law."

### DEATH PLANT OF JAVA.

A magnificent kali mujah or, death plant, of Java, has been recently received at Savannah. This specimen, which is the only living one that has ever been brought to this country, was sent Mrs. Black by her brother, Jerome Hendricks, who went out as a missionary to the island. The kali mujah is found only in the volcanic districts of Java and Sumatra, and then but rarely. It grows from two to three and a half feet in height, with long, slender stems, armed with thorns nearly an inch long, and covered with broad satin-smooth leaves of a heart shape and of a delicate emerald on one side and blood red, streaked with cream, on the other.

The flowers of the death plant are large, milk-white, and cup-like, being about the size and depth of a large coffee cup, and having the rim guarded by fine brier-like thorns. The peculiarity of the plant lies in these flowers, which, beautiful as they are, distill continually a deadly perfume so powerful as to overcome, if inhaled any length of time, a full-grown man, and killing all forms of insect life approaching it. The perfume, though more pungent, is as sickeningly sweet as chloroform, which it greatly resembles in effect, producing insensibility, but convulsing at the same time the muscles of the face, especially those about the mouth and eyes, drawing the former up into a grin. An inhalation is followed by violent headache and ringing in the ears, which gives way to a temporary deafness, often total while it lasts.

Other plants seem to shun the kali mujah, which might be termed the Ishmael of the vegetable kingdom, for it grows isolated from every other form of vegetation, though the soil about it may be fertile. All insects and birds instinctively seem to avoid all contact with it, but when accidentally approaching it have been observed to drop to the earth, even when as far from it as three feet, and, unless at once removed, soon died, evincing the same symptoms as when etherized.

Mr. Hendricks who writes describing how he secured the specimen sent his sister, says he discovered it first by seeing a bird of paradise he was endeavoring to capture alive, fall stunned by the deadly odor of the kali mujah, and on examining the plant, though warned by the natives to let it alone, himself experienced the headache and convulsions which are its invariable results.

### ARMY SUPPLIES.

The contract for supplying baking powder to the U. S. Army, bids for which were recently opened in New York, has been awarded to the Cleveland Baking Powder Co. Before the award was made the different baking powders offered were submitted to a thorough analysis, with the sanction of Commissary General DuBary.

### WONDERS OF THE SEA.

The sea occupies three-fifths of the surface of the earth. At the depth of about 3,500 feet waves are not felt. The temperature is the same, varying only a trifle from the ice of the pole to the burning sun of the equator. A mile down the water has a pressure of over a ton to the square inch. If a box 6 feet deep were filled with sea water allowed to evaporate under the sun, there would be 2 inches of salt left on the bottom. Taking the average depth of the ócean to be three miles, there would be a layer of pure salt 230 feet thick on the bed of the Atlantic. The water is colder at the bottom than at the surface. In the many bays on the coast of Norway the water often freezes at the bottom before it does above. Waves are very deceptive. To look at them in a storm one would think the water traveled. The water stays in the same place, but the motion goes on. Sometimes in storms these waves are 40 feet high, and travel fifty miles an hour—more than twice as fast as the swiftest steamship. The distance from valley to valley is generally fifteen times the height, hence a wave 5 feet high will extend over 75 feet of water. The force of the sea dashing on Bell Rock is said to be seventeen tons for each square yard. Evaporation is a wonderful power in drawing the water from the sea. Every year a layer of the entire sea, 14 feet thick, is taken up into the clouds. The winds bear their burden into the land, and the water comes down in rain upon the fields, to flow back at last through rivers. The depth of the sea presents an interesting problem. If the Atlantic were lowered from 6,564 feet, the distance from shore to shore would be half as great, or 1,500 miles. If lowered a little more than three miles, say 19,680 feet, there would be a road of dry land from Newfoundland to Ireland. This is the plain on which the great Atlantic cables were laid. The Mediterranean is comparatively shallow. A drying up of 660 feet would leave three different seas, and Africa would be joined with Italy.

